# Universality of Citation Distributions for Academic Institutions and Journals

**DOI:** 10.1371/journal.pone.0146762

**Published:** 2016-01-11

**Authors:** Arnab Chatterjee, Asim Ghosh, Bikas K. Chakrabarti

**Affiliations:** 1 Condensed Matter Physics Division, Saha Institute of Nuclear Physics, 1/AF Bidhannagar, Kolkata 700064, India; 2 Department of Computer Science, Aalto University School of Science, P.O. Box 15400, FI-00076 AALTO, Finland; 3 Economic Research Unit, Indian Statistical Institute, 203 B. T. Road, Kolkata 700108, India; Max Planck Society, GERMANY

## Abstract

Citations measure the importance of a publication, and may serve as a proxy for its popularity and quality of its contents. Here we study the distributions of citations to publications from individual academic institutions for a single year. The average number of citations have large variations between different institutions across the world, but the probability distributions of citations for individual institutions can be rescaled to a common form by scaling the citations by the average number of citations for that institution. We find this feature seems to be universal for a broad selection of institutions irrespective of the average number of citations per article. A similar analysis for citations to publications in a particular journal in a single year reveals similar results. We find high absolute inequality for both these sets, Gini coefficients being around 0.66 and 0.58 for institutions and journals respectively. We also find that the top 25% of the articles hold about 75% of the total citations for institutions and the top 29% of the articles hold about 71% of the total citations for journals.

## Introduction

Statistical physics tells us that systems of many interacting dynamical units collectively exhibit a behavior which is determined by only a few basic dynamical features of the individual units and of the embedding dimension but independent of all other details. This feature which is specific to critical phenomena, like in continuous phase transitions, is known as universality [1]. There is enough empirical evidence that a number of social phenomena are characterized by simple emergent behavior out of the interactions of many individuals. In recent years, a growing community of researchers have been analyzing large-scale social dynamics to uncover universal patterns and also trying to propose simple microscopic models to describe them, similar to the minimalistic models used in statistical physics. These studies have revealed interesting patterns and behaviors in social systems, e.g., in elections [2–4], growth in population [5] and economy [6], income and wealth distributions [7], financial markets [8], languages [9], etc. (see Refs. [10, 11] for reviews).

Academic publications (papers, books etc.) form an unique social system consisting of individual publications as entities, containing bibliographic reference to other older publications, and this is commonly termed as *citation*. The number of citations is a measure of the importance of a publication, and serve as a proxy for the popularity and quality of a publication. There has already been a plethora of empirical studies on citation data [11], specifically on citation distributions [12–15] of articles, time evolution of probability distribution of citation [16–18], citations for individuals [19] and even their dynamics [20], and the modeling efforts on the growth and structure of citation networks have produced a huge body literature in network science concerning scale-free networks [21–23], and long-time scientific impact [24].

The bibliometric tool of citation analysis is becoming increasingly popular for evaluating the performance of individuals, research groups, institutions as well as countries, the outcomes of which are becoming important in case of offering grants and awards, academic promotions and ranking, as well as jobs in academia, industry and otherwise. Since citations serve as a crucial measure for the importance and impact of a research publication, its precise analysis is extremely important. Annual citations and impact factor of journals are of key interest, primarily from the point of view of journals themselves, and secondarily from the perspective of authors who publish their papers in them. Wide distributions of both annual citations and impact factors are quite well studied [25–27]. It is quite usual to find that some publications do better than others due to the inherent heterogeneity in the quality of their content, the gross attention on the field of research, the relevance to future work and so on. Thus different publications gather citations in time at different rates and result in a broad distribution of citations. In 1957, Shockley [12] claimed that the scientific publication rate is dictated by a lognormal distribution, while a later evidence based on analysis of records for highly cited physicists claim that the citation distribution of individual authors follow a stretched exponential [13]. However, an analysis of data from ISI claims that the tail of the citation distribution of individual publications decays as a power law with an exponent close to 3 [14], while a rigorous analysis of 110 years of data from Physical Review concluded that most part of the citation distribution fits remarkably well to a lognormal [28]. The present consensus lies with the fact that while most part of the distribution does fit to a lognormal, the extreme tail fits to a power law [29].

It has been shown earlier [15] that the distribution of citations *c* to papers within a discipline has a broad distribution, which is universal across broad scientific disciplines, using a relative indicator *c*_*f*_ = *c*/〈*c*〉, where 〈*c*〉 is the average citation within a discipline. However, it has also been shown later that this universality is not absolutely guaranteed [30]. Subsequent work on citations [31, 32] and impact factors [33] has revealed interesting patterns of universality, some alternative methods have been proposed [34] and there are also interesting work on citation biases [32]. Some studies [35, 36] also report on the possible lack of universality in the citation distribution at the level of articles. A rigorous and detailed study on the citation distributions of papers published in 2005–2008 for 500 institutions [37] reveals that using the analysis Ref. [15], universality condition is not fully satisfied, but the distributions are found to be very similar. There have also been studies at the level of countries in the same direction [38].

In this article, we focus on citations received by individual (i) academic institutions and (ii) academic journals. We perform the analysis primarily for all articles and reviews, as well as all citable documents. While institutions can vary in their quality of scientific output measurable in terms of total number of publications, total citations etc., here we show for the first time that irrespective of the institution’s scientific productivity, ranking and research impact, the probability *P*(*c*) that the number of citations *c* received by a publication is a broad distribution with an universal functional form. In fact, using a relative indicator *c*_*f*_ = *c*/〈*c*〉, where 〈*c*〉 is the average number of citations to articles published by an institution in a certain year, we show that the effective probability distribution function that an article has *c* citations has the same mathematical form. We present evidence for the fact that this holds roughly across time for most institutions irrespective of the scientific productivity of the institution considered. When we carry out a similar analysis on journals, we find similar results. The scaled distributions fit to a lognormal distribution for most of their range. Again, we find that these features roughly hold across time and across journals within the same class. The largest citations for academic institutions as well as the journals seem to fit well to a power law. We also present evidence that each of these sampled groups—institutions, and journals are distinct with the absolute measure of inequality as computed from their distribution functions, with high absolute inequality for both these sets, the Gini coefficients being around 0.66 and 0.58 for institutions and journals respectively. We also find that the top 25% of the articles fetch about 75% of the total citations for institutions and the top 29% of the articles fetch about 71% of the total citations for journals.

## Methods

### Data

We collected data from 42 academic institutions across the world. Institutions were selected such that they produce considerable amount of papers (typically 200 or more) so that reasonable statistics could be obtained. However, there were exceptions for certain years for particular institutions. All papers published with at least one author with the institution mentioned as affiliation were collected. This was done for 4 years—1980, 1990, 2000, 2010. We also selected 30 popular academic journals across physics, chemistry, biology and medicine. However, for some journals, only 3 years of data could be collected, since they were launched after 1980. The citable papers considered in this study are articles and reviews, although we compare the results with the same analysis done on all citable documents.

## Results

We study the data of number of citations to publications from different years, from ISI Web of Science [39] for several (i) academic institutions (research institutes and universities) and (ii) popular journals. It is to be noted that citations to individual publications arrive from any publication indexed in ISI Web of Science and does not mean only internal citations within the journal in which it is published. We analyzed data of science publications from 42 academic institutions and 30 popular journals. We recorded the data for the number of papers published, the total number of citations to each of the publications, for a few years (1980, 1990, 2000, 2010 for most cases). Since citations grow with time, we have studied publications which are at least 4 years old (from 2010) or more (1980, 1990, 2000) to rule out any role of transients. We also collect data from academic institutions and journals which have a comparatively large number of publications, so as to produce good statistics, and minimize the effects of aberration that can result from fluctuations of the quantities measured from small data sets.

### Citations for academic institutions

**Fig 1:**
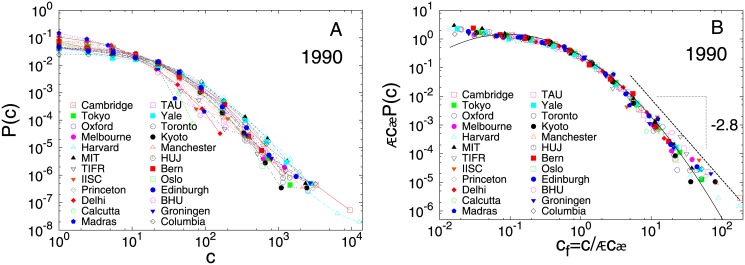
Probability distribution of citation for academic institutions for 1990 (unscaled and rescaled). (A) Probability distribution *P*(*c*) of citations *c* to publications from 1990 for several academic institutions. (B) The same data rescaled by average number of citations 〈*c*〉. The data for different institutions seem to follow the same scaling function. It fits very well to a lognormal function for most of its range, with *μ* = −0.73 ± 0.02, *σ* = 1.29 ± 0.02. The largest citations do not follow the lognormal behavior, and seem to follow a power law: *c*^−*α*^, with *α* = 2.8 ± 0.2.

**Fig 2:**
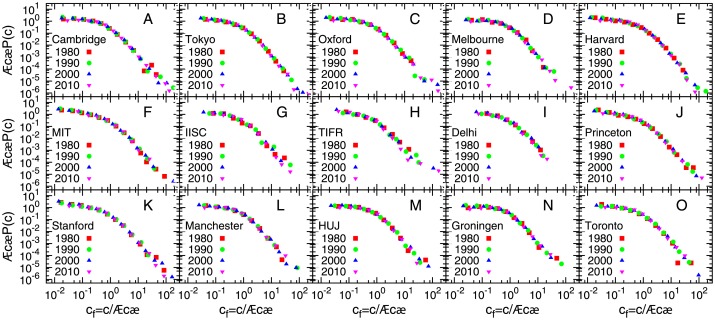
Rescaled probability distributions of citation for several academic institutions for different years. Probability distribution *P*(*c*) of citations *c* rescaled by average number of citations 〈*c*〉 to publications from 4 different years (1980, 1990, 2000, 2010) for several academic institutions. For any institution, the data for different years seem to follow the same distribution.

We collected citation data until date for all articles and reviews from a particular year (e.g. 1980, 1990, 2000, 2010). For each year, the probability distribution *P*(*c*) of citations *c* for an academic institution was observed to be broad. For instance, Fig 1A shows the plot of *P*(*c*) vs. *c* for various institutions for publications from 1990. We rescaled the absolute value of citation for each year by the average number of citations per publication 〈*c*〉, and plotted this quantity *c*_*f*_ = *c*/〈*c*〉 against the adjusted probability 〈*c*〉*P*(*c*) (Fig 1B) (see similar plots for 1980 and 2000 in Fig A in S1 File). We remarkably find that the distributions collapse into an universal curve irrespective of the wide variation in the academic output of the different institutions. The scaling collapse is good for more than 3 decades of data and over 5 orders of magnitude. The average number of papers, total citations and the average number of citations per publication are shown in Table C in S1 File. The rescaled curves fit well to a lognormal

$$F\left(x\right)=\frac{1}{x\sigma \sqrt{2\pi }}\exp\left[-\frac{{\left(\log x-\mu \right)}^{2}}{2\sigma^{2}}\right]$$ (1)

with *μ* = −0.73 ± 0.02 with *σ* = 1.29 ± 0.02, for a considerable range of the distribution. However, if one fits a lognormal distribution to individual sets, the range of parameters are quite narrow, *μ* lies in the range −1.2 to −0.6, while *σ* lies in the range 1.0 to 1.6. The fitting were performed using a least square fitting routine. For lowest values of the abscissa, seems to follow 〈*c*〉*P*(*c*) → *const* or slowly growing, as *c* → 0. However, the largest citations deviate from the lognormal fit and are better described according to *P*(*c*)∼*c*^−*α*^, with *α* = 2.8 ± 0.2 (see Table F in S1 File for exponents for other years). The power law exponent has been estimated using the maximum likelihood estimate method (MLE) [40]. In order to investigate if the distributions *P*(*c*) for different institutes vary with time, we plot the same for each institution for several years. The rescaled plots show scaling collapse indicating that although the average citations vary over years, the form of the distribution function remain roughly invariant, when scaled with the average number of citations. Fig 2 shows the plot for 1990. To check if this also holds for time-aggregated data, we collected citations for all papers published during the period 2001–2005 for the same set of institutions, and repeated the above analysis (see Fig B in S1 File).

### Citations for journals

**Fig 3:**
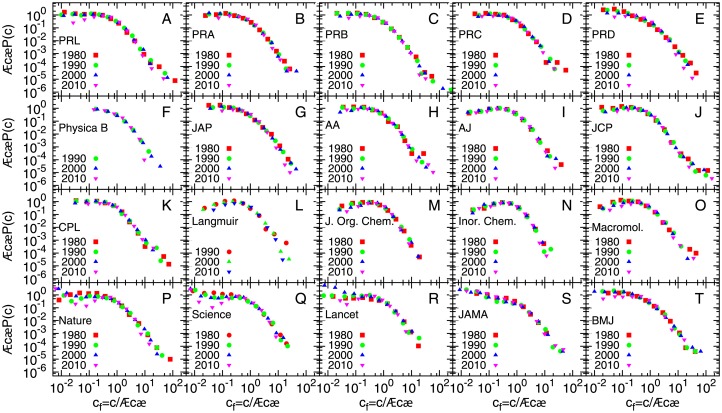
Rescaled probability distributions of citation for academic journals for different years. Probability distribution *P*(*c*) of citations *c* rescaled by average number of citations 〈*c*〉 to publications from from 4 different years (1980, 1990, 2000, 2010) for several academic journal. For any journal, the data for different years seem to follow the same distribution.

**Fig 4:**
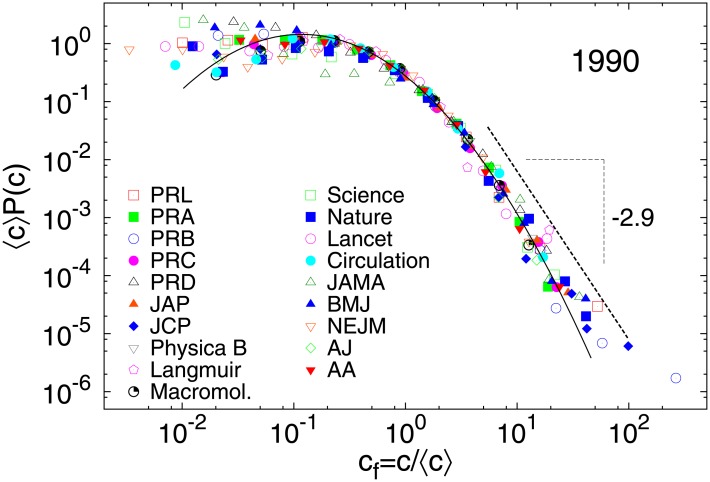
Rescaled probability distributions of citation for several journals for 1990. Probability distribution *P*(*c*) of citations *c* rescaled by average number of citations 〈*c*〉 to publications from 1990 for several academic journals. The scaled distribution fucntion fits to a lognormal function with *μ* = −0.75 ± 0.02, *σ* = 1.18 ± 0.02, while 〈*c*〉*P*(*c*) → *const*. as *c*/〈*c*〉 → 0 for the lower range of *c*. The largest citations fit well to a power law: *c*^−*α*^, with *α* = 2.9 ± 0.3.

We collected citation data until date for all articles and reviews in individual journals for several years (e.g. 1980, 1990, 2000, 2010 etc.). For each year, the probability distribution *P*(*c*) of citations *c* was again observed to be broad. As in the case of institutions, we plotted *c*_*f*_ = *c*/〈*c*〉 against the adjusted probability 〈*c*〉*P*(*c*) (Fig 3). For a particular journal, it is observed that the curves follow similar distributions over years although the average number of papers, total citations and hence the average number of citations vary (see Table D in S1 File for details). Further, we plot the same quantity for a particular year for different journals (see Fig 4 for 1990), and find that the curves roughly collapse into an single curve irrespective of the wide variation in the output of the different journals. The bulk of the rescaled distribution fits well to a lognormal form with *μ* = −0.75 ± 0.02 and *σ* = 1.18 ± 0.02, as was observed in case of institutions, while the largest citations fit better to a power law *P*(*c*)∼*c*^−*α*^, with *α* ≈ 2.9 ± 0.3 (see Table F in S1 File for exponents for other years). To check if this also holds for time-aggregated data, we collected citations for all papers published during the period 2001–2005 for the same set of journals and repeated the analysis (see Fig D in S1 File).

However, we observed that if we consider all citable documents, two distinct classes of journals emerge according to the shape of the distributions to which the curves collapse. The first group is a *General* class, for which most of the distribution fits well to a lognormal function even quite well for the lowest values of the abscissa. This is similar to what is observed for all journals if we consider only articles and reviews. The other group, which we call the *Elite* class (Fig E in S1 File) is also broadly distributed but has a distinct and faster monotonic decay compared to the *General* class, where 〈*c*〉*P*(*c*)∼(*c*/〈*c*〉)^−*b*^, i.e., *P*(*c*)∼*c*^−*b*^/〈*c*〉^(1−*b*)^ with *b* ≃ 1. This divergence at the lowest values of citations also indicate that the *Elite* journals have a larger proportion of publications with less number of citations although their average number of citations 〈*c*〉 is larger than those for the general class. However, for both the above classes, the largest citations still follow a power law *P*(*c*)∼*c*^−*α*^, with *α* ≈ 2.8 ± 0.4 for the *General* class and *α* ≈ 2.7 ± 0.6 for the *Elite* class. We reason for such a behavior in the *Elite* journal class is because of a large fraction *f*_0_ of uncited documents. If we consider only articles and reviews, *f*_0_ is usually 2–10%. Considering all citable documents, this fraction does not change appreciably for the *Elite* class of journals, and can be anything in the range 25–80% (see values of *f*_0_ in Table E in S1 File), and are primarily in the category of news, correspondence, editorials etc. Such documents in the *General* class is either absent or are very few. We are able to find at least 7 journals (see Fig E and Table E in S1 File) in the *Elite* class while most of others belong to the *General* class.

The power law tail in all distribution suggests that the mechanism behind the popularity of the very highly cited papers is a ‘rich gets richer’ phenomena [22, 41, 42] (see Fig C in S1 File for 1980 and 2000).

### Justification for using *c*_*f*_ = *c*/〈*c*〉

**Fig 5:**
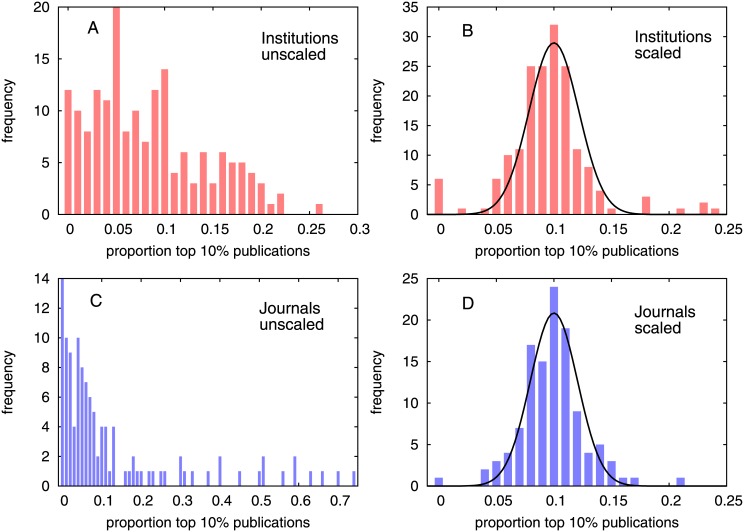
Percentage of publications of each institution that appear in the top 10% of the global rank. Histograms for the percentage of publications of each institution that appear in the top 10% of the global rank, computed from the (A) unscaled and (B) scaled data. Same for the percentage of publications of each journal that appear in the top 10% of the global rank, computed from the (C) unscaled and (D) scaled data. A normal distribution fit to the scaled data gives *σ*_*z*_ = 2.15 ± 0.08 for institutions and *σ*_*z*_ = 2.05 ± 0.08 for journals.

Following Ref. [15], we rank all articles belonging to different institutions according to *c* and *c*_*f*_. We then compute the percentage of publications of each institution that appear in the top *z*% of the global rank. The percentage for each should be around *z*% with small fluctuations if the ranking is good enough. The same is performed for journals. When ranking is done according to unnormalized citations *c* then the frequency distribution of *z*% of papers is wide. However, if the ranking is done according to normalized citations *c*_*f*_, then the frequency distribution is much narrow. For example, we show the results for institutions and journals in Fig 5 if *z* = 10%. Assuming that articles are uniformly distributed on the rank axis, the expected average bin height must be z% with a standard deviation given by

$$\sigma_{z}=\sqrt{\frac{z(100-z)}{N}\sum_{i=1}^{N}\frac{1}{N_{i}}}.$$ (2)

where *N* is the number of entries (institutions or journals) and *N*_*i*_ is the number of papers for the *i*-th institution or journal. For institutions, when the ranking is done according to *c*_*f*_ we observe that the theoretically calculated value (from above equation) of *σ*_*z*_ is 1.25 compared to 2.15 ± 0.08 as computed directly from the fitting, while if the ranking was done according to *c*, *σ*_*z*_ is 6.29. Similarly for journals, *σ*_*z*_ computed from the above equation is 1.04 compared to 2.05 ± 0.08 as computed from the fitting, while if the ranking was done according to *c*, *σ*_*z*_ is 17.73. This indicates that *c*_*f*_ is indeed an unbiased indicator, as seen earlier [15, 30].

### Measuring inequality

We calculate absolute measures of inequality like the commonly used Gini index [43] as well as the *k*-index [44, 45] which tells us that the top cited 1−*k* fraction of papers have *k* fraction of citations, and we report in Tables C, D, E in S1 File. For academic institutions, Gini index *g* = 0.67 ± 0.10 and *k* = 0.75 ± 0.04, which means around 75% citations come from the top 25% papers. For journals, *g* = 0.58 ± 0.15, *k* = 0.71 ± 0.08 which means about 71% citations come from the top 29% papers.

**Fig 6:**
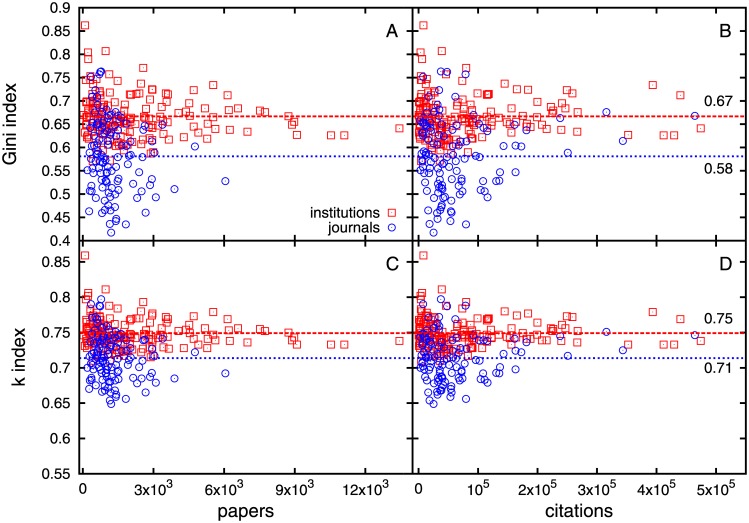
Gini and *k* indices with number of papers and citations. Variation of Gini and *k* indices with number of papers and citations for academic institutions and journals. For larger number of papers or citations, the values seem to fluctuate less or converge around the mean values $\bar{g}$ and $\bar{k}$ respectively. For academic institutions, the values are $\bar{g}\approx 0.67$ for Gini and $\bar{k}\approx 0.75$, while for the journals, the values are $\bar{g}\approx 0.58$ and $\bar{k}\approx 0.71$.

We further note that Gini and *k* indices fluctuate less around respective mean values $\bar{g}$ and $\bar{k}$ as the number of articles and number of citations become large (Fig 6). For academic institutions, the values are $\bar{g}\approx 0.66$ for Gini and $\bar{k}\approx 0.75$. For journals, the values are $\bar{g}\approx 0.58$ and $\bar{k}\approx 0.71$.

## Discussions

In this article we analyze whether the citations to science publications from academic institutions (universities, research institutes etc.) as well as journals are distributed according to some universal function when rescaled by the average number of citations. For institutions, it seems to fit roughly to a log-normal function. The largest citations, however, deviate from the lognormal fit, and follow a power law decay. This rough universality claim is an interesting feature, since for institutions, the quality of scientific output measurable in terms of the total number of publications, total citations etc. vary widely across the world as well as in time. Nevertheless, the way in which the number of papers with a certain number of citations is distributed is quite similar [37], seems to be quite independent of the quality of production/output of the academic institution. Although there has been claims that the form of the distribution of citations for different scientific disciplines are the same [15], albeit deviations [30], it is also true that each discipline is characterized by a typical average number of citations 〈*c*〉_*d*_. As a matter of fact, that different institutions have a varying strength of publication contribution towards different disciplines makes the issue of obtaining a universal function for the resulting (effective) distribution of citations (for the institution) quite nontrivial. In other words, different academic institutions have a variety in the strength of their academic output, in terms of variation of representations across different disciplines and the amount of citations gathered. This does not necessarily guarantee that the universality which has been already reported across disciplines [15] will still hold when one looks at data from different institutions, rest aside the counter claims about lack of universal character [30] for citation distribution across distinct disciplines. There are already critical studies on the citation distribution of universities [37] using larger data sets, which raises issues on the nature of universality.

We observe similar features for academic journals—the bulk of the probability distribution fitting reasonably well to a lognormal while the highest cited papers seem to fit well to a power law decay with a similar exponent (2.7–3.0). We note that the exponents are consistently less than 3, the exponent of the full citation distribution [14], which is due to the fact that our data are very small subsets, which fall short of catching the correct statistical behavior of all of the highest cited papers.

Our results indicate that dividing citation counts by their average indeed helps to get closer to universal citation distributions. However, the results also indicate that, even after such a rescaling, *σ*_*z*_ are substantially larger than the theoretical values −1.25 compared to 2.15 ± 0.08 while it is 6.29 for unscaled data for institutions and 1.04 compared to 2.05 ± 0.08 while it is 17.73 for unscaled data for journals. This indicates that the universality is not very strong, and holds only in an approximate sense. Ref. [37] shows similar evidence for institutions, claiming the absence of universality but pointing out the similarity between the distributions. Another previous study [30] on different fields of science also reported that this universality claim does not hold very well for all fields.

We further note that the inequality in the distribution of citations of institutions and journals differ quantitatively. As the number of papers and citations increase, the absolute measures of inequality like Gini and *k* indices seem to converge to different values for the above two sets. The values of Gini index are 0.66 and 0.58 for institutions and journals respectively. The *k* index values suggest that the top 25% of the articles hold about 75% of the total citations for institutions and the top 29% of the articles hold about 71% of the total citations for journals.

## Supporting Information

S1 File Supporting Information: Universality of citation distributions for academic institutions and journals. (PDF)
