# Correction: Universality of Citation Distributions for Academic Institutions and Journals

**DOI:** 10.1371/journal.pone.0148863

**Published:** 2016-02-05

**Authors:** The PLOS ONE staff

Figs 1, 2 and 3 are incorrect. The x and y axes of each figure are mislabeled. The publisher apologizes for the error. The authors have provided a corrected version here.

**Fig 1:**
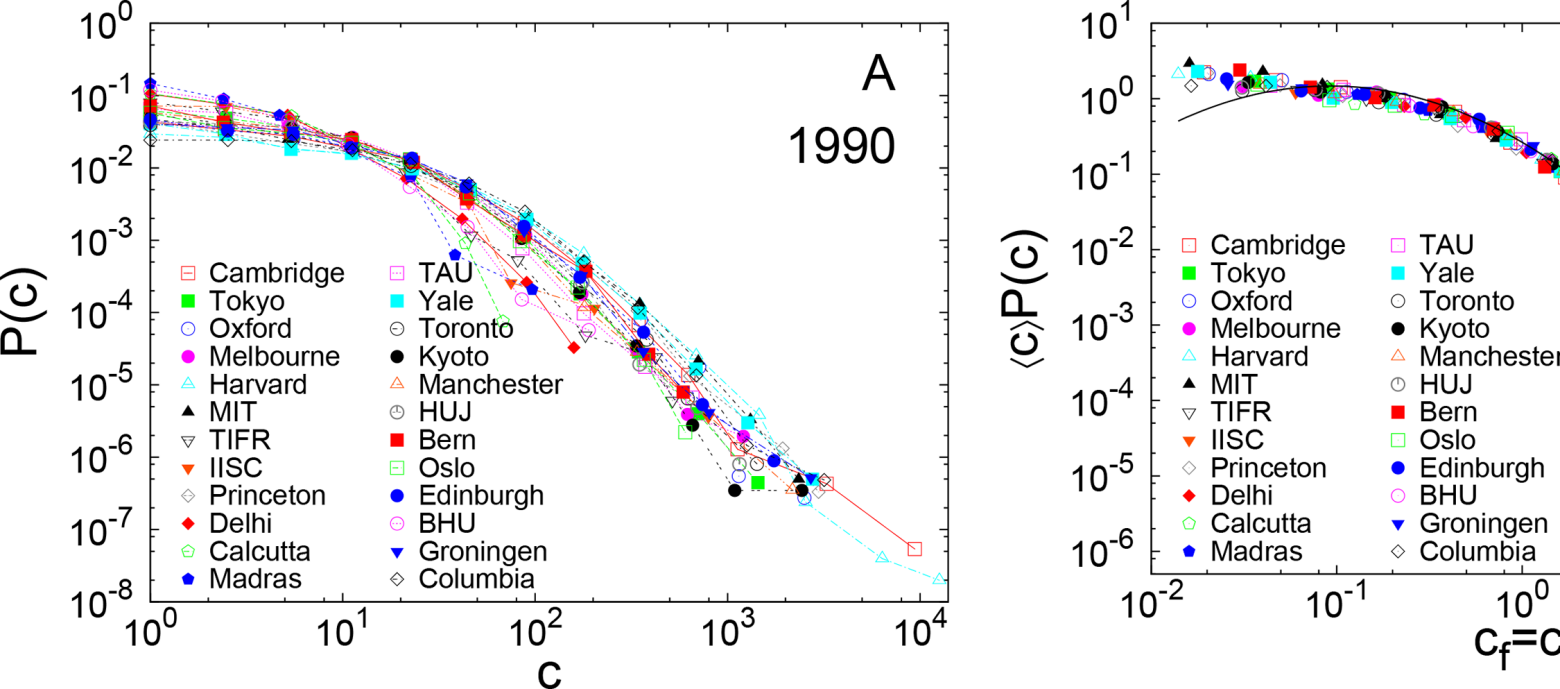
Probability distribution of citation for academic institutions for 1990 (unscaled and rescaled). (A) Probability distribution *P*(c) of citations c to publications from 1990 for several academic institutions. (B) The same data rescaled by average number of citations 〈c〉. The data for different institutions seem to follow the same scaling function. It fits very well to a lognormal function for most of its range, with μ = −0.73 ± 0.02, σ = 1.29 ± 0.02. The largest citations do not follow the lognormal behavior, and seem to follow a power law: c−α, with α = 2.8 ± 0.2.

**Fig 2:**
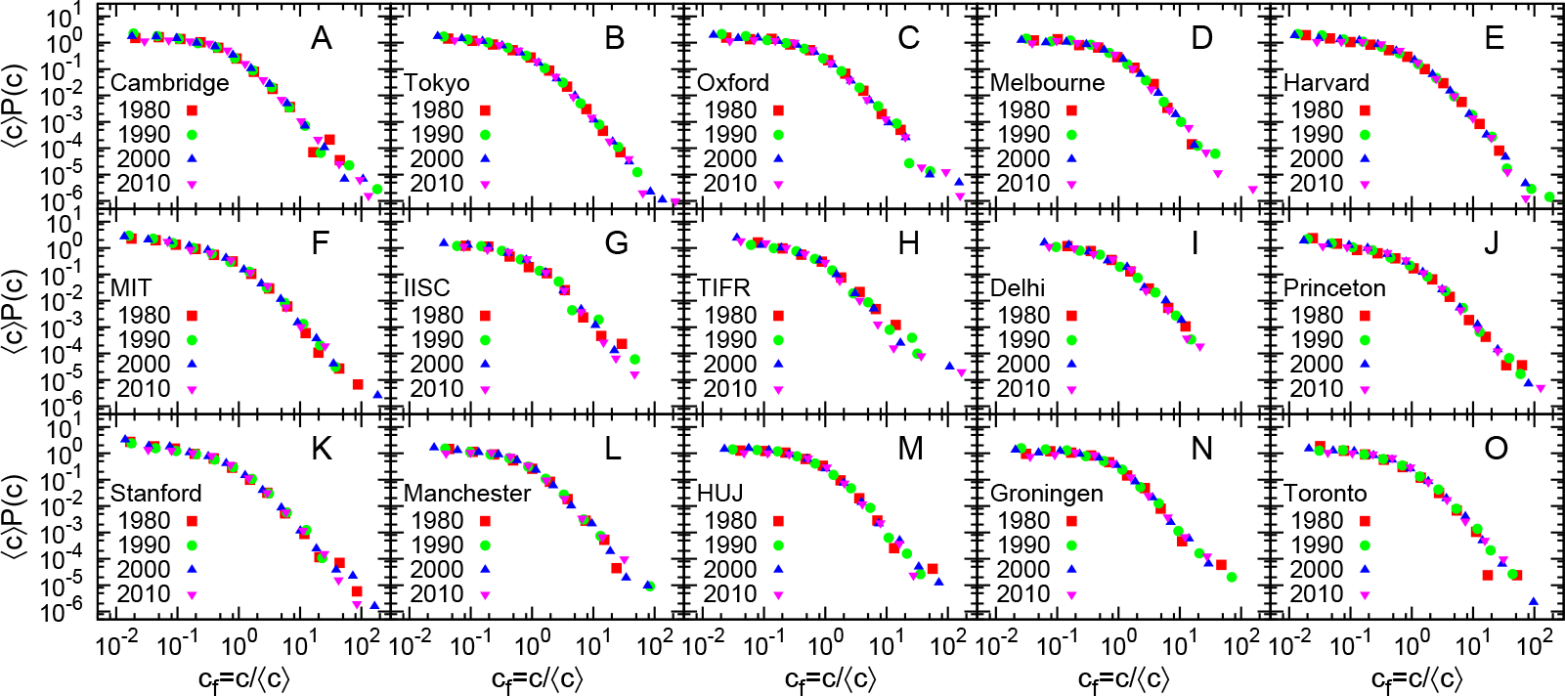
Rescaled probability distributions of citation for several academic institutions for different years. Probability distribution *P*(c) of citations c rescaled by average number of citations 〈c〉 to publications from 4 different years (1980, 1990, 2000, 2010) for several academic institutions. For any institution, the data for different years seem to follow the same distribution.

**Fig 3:**
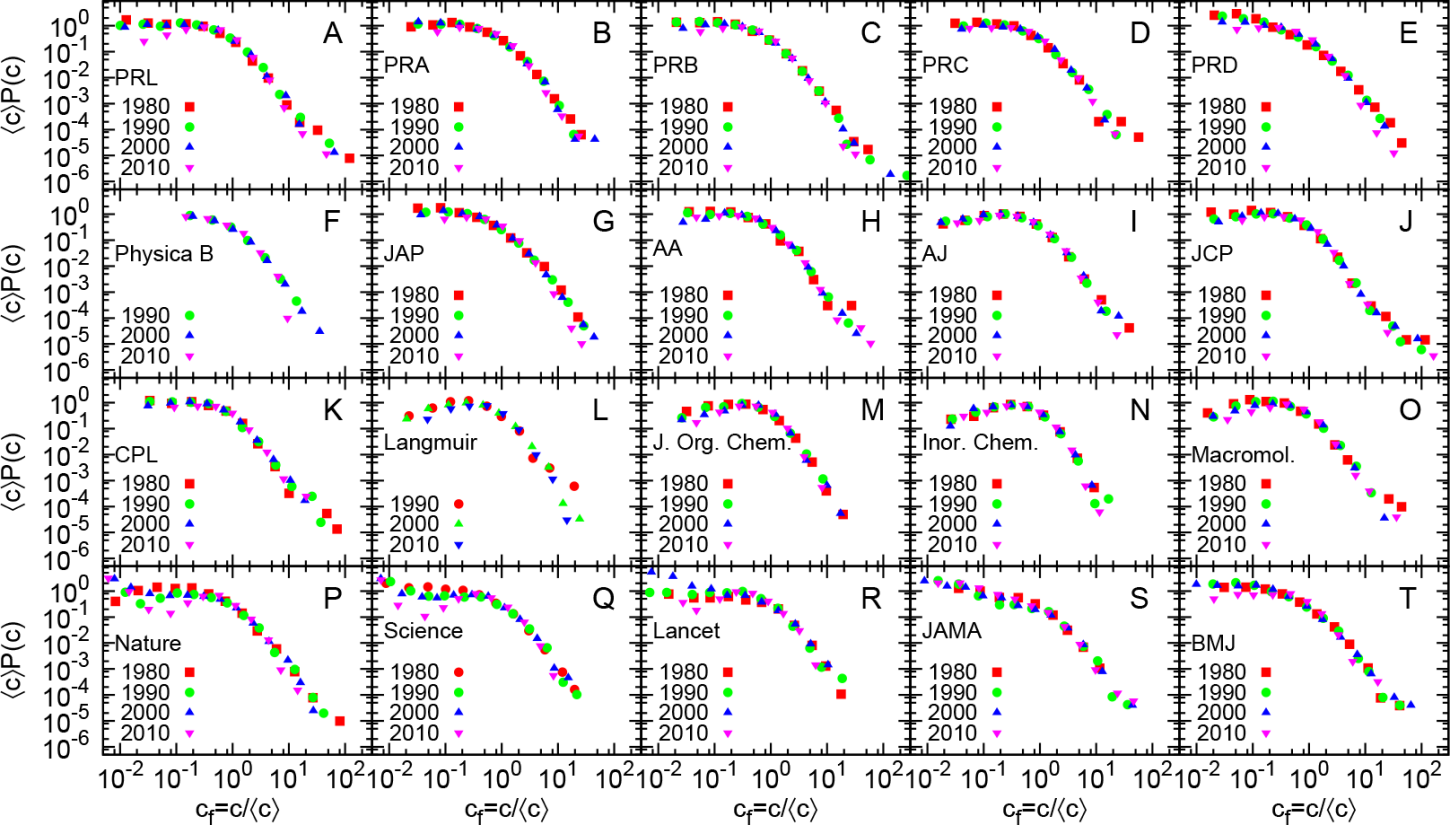
Rescaled probability distributions of citation for academic journals for different years. Probability distribution *P*(c) of citations c rescaled by average number of citations 〈c〉 to publications from from 4 different years (1980, 1990, 2000, 2010) for several academic journal. For any journal, the data for different years seem to follow the same distribution.
