# Supplementary material for: Universality of Citation Distributions for Academic Institutions and Journals
Source: PLoS One. 2016 Jan 11;11(1):e0146762. doi: 10.1371/journal.pone.0146762 (PMC4709109; doi:10.1371/journal.pone.0146762)
Supplement: S1 File — (PDF) [file pone.0146762.s001.pdf]

Supporting Information: Universality of citation distributions for academic institutions and journals

Arnab Chatterjee,[1] Asim Ghosh,[1,2] and Bikas K Chakrabarti[1,3]

[1] *Condensed Matter Physics Division, Saha Institute of Nuclear Physics, 1/AF Bidhannagar, Kolkata 700064 India.*
[2] *Department of Computer Science, Aalto University School of Science, P.O. Box 15400, FI-00076 AALTO, Finland.*
[3] *Economic Research Unit, Indian Statistical Institute, 203 B. T. Road, Kolkata 700108, India.*

S1 TABLE A: **Abbreviations for institutions.** Table showing abbreviations used for academic institutions.

Abbreviation	Full Name of University / Institute	Abbreviation	Full Name of University / Institute
Bern	University of Bern	Kyoto	Kyoto University
BHU	Banaras Hindu University	Landau Inst.	Landau Institute for Theoretical Physics
Bordeaux	University of Bordeaux	Leiden	Leiden University
Boston	Boston University	Leuven	University of Leuven – KU Leuven
Bristol	University of Bristol	Madras	University of Madras
Buenos Aires	University of Buenos Aires	Manchester	The University of Manchester
Calcutta	University of Calcutta	Melbourne	The University of Melbourne
Caltech	California Institute of Technology	MIT	Massachusetts Institute of Technology
Cambridge	University of Cambridge	Osaka	Osaka University
Chicago	The University of Chicago	Oslo	University of Oslo
Cologne	University of Cologne	Oxford	University of Oxford
Columbia	Columbia University	Princeton	Princeton University
Delhi	University of Delhi	SINP	Saha Institute of Nuclear Physics
Edinburgh	The University of Edinburgh	Stanford	Stanford University
Gottingen	University of Göttingen	Stockholm	Stockholm University
Groningen	University of Groningen	TAU	Tel Aviv University
Harvard	Harvard University	TIFR	Tata Institute of Fundamental Research
Heidelberg	Heidelberg University	Tokyo	The University of Tokyo
Helsinki	University of Helsinki	Toronto	University of Toronto
HUJ	The Hebrew University of Jerusalem	Yale	Yale University
IISC	Indian Institute of Science	Zurich	University of Zurich

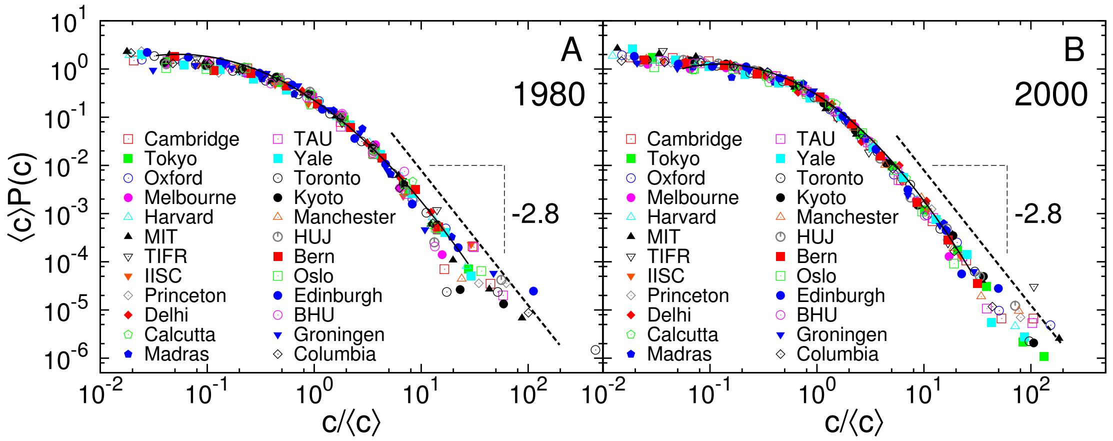

S1 Fig. A: **Probability distribution of citations for academic institutions for 1980, 2000.**
Probability distribution $P(c)$ of citations c rescaled by average number of citations $\langle c \rangle$ to publications from 2 different years (1980, 2000) for several academic institutions. Most of the range of the data fit well to a lognormal function with $\mu = -0.98 \pm 0.02$, $\sigma = 1.40 \pm 0.03$ for 1980 and $\mu = -0.61 \pm 0.02$, $\sigma = 1.21 \pm 0.02$ for 2000. but the highest citations fit to power law, with $\alpha = 2.8 \pm 0.2$ for 1980 and $\alpha = 2.8 \pm 0.1$ for 2000.

S1 TABLE B: **Abbreviation of journals, impact factors.**
Table showing abbreviations for journals, and their 2010 Impact factor [1].

Abbreviation	Full Name of Journal	2010 Impact Factor
AA	Astronomy & Astrophysics	4.425
AJ	The Astrophysical Journal	6.063
Biochemistry	Biochemistry	3.226
CPL	Chemical Physics Letters	2.282
Eur. J. Biochem./ FEBS Journal	European Journal of Biochemistry (before 2005) FEBS Journal (2005 onwards)	3.129
Inor. Chem.	Inorganic Chemistry	4.326
JAP	Journal of Applied Physics	2.079
JCP	Journal of Chemical Physics	2.921
JMMM	Journal of Magnetism and Magnetic Materials	1.690
J. Org. Chem.	Journal of Organic Chemistry	4.002
JPA	Journal of Physics A: Mathematical and General (before 2007) Journal of Physics A: Mathematical and Theoretical (2007 onwards)	1.641
Langmuir	Langmuir	4.269
Macromol.	Macromolecules	4.838
Physica A	Physica A	1.522
Physica B	Physica B	0.856
Physica C	Physica C	1.415
PRA	Physical Review A	2.861
PRB	Physical Review B	3.774
PRC	Physical Review C	3.416
PRD	Physical Review D	4.964
PRE	Physical Review E	2.352
PRL	Physical Review Letters	7.622
Tetrahedron	Tetrahedron	3.011
BMJ	British Medical Journal	13.471
Circulation	Circulation	14.432
JAMA	The Journal of the American Medical Association	30.011
Lancet	Lancet	33.633
Nature	Nature	36.104
NEJM	The New England Journal of Medicine	53.486
Science	Science	31.377

[1] Thompson Reuters, Journal Citation Reports, retreived March, 2013. http://admin-apps.webofknowledge.com/JCR/JCR?RQ=HOME.

S1 TABLE C: **Academic institutions: papers, citations etc.**

Table showing data for number of papers, total number of citations, average citation per paper $\langle c \rangle$, Gini (g) index, k index, and the fraction of uncited papers f_0 from different institutions for several years.

Institutions	Year	Papers	Citations	$\langle c \rangle$	g	k	f_0
Bern	1980	597	12029	20.15	0.705	0.767	0.162
	1990	735	24468	33.29	0.684	0.759	0.076
	2000	1145	47621	41.59	0.615	0.726	0.040
	2010	1897	46576	24.55	0.621	0.726	0.034
BHU	1980	454	2739	6.03	0.681	0.757	0.244
	1990	411	3935	9.57	0.708	0.767	0.212
	2000	371	5271	14.21	0.635	0.737	0.105
	2010	862	12239	14.20	0.628	0.728	0.084
Bordeaux	1980	412	7873	19.11	0.685	0.762	0.119
	1990	671	14802	22.06	0.671	0.750	0.088
	2000	1089	37154	34.12	0.647	0.740	0.055
	2010	1903	41883	22.01	0.606	0.723	0.050
Boston	1980	628	29097	46.33	0.661	0.749	0.065
	1990	1068	64787	60.66	0.690	0.759	0.050
	2000	1753	107119	61.11	0.657	0.744	0.036
	2010	2829	89245	31.55	0.620	0.729	0.031
Bristol	1980	595	22621	38.02	0.599	0.717	0.054
	1990	936	35370	37.79	0.642	0.737	0.063
	2000	1771	84466	47.69	0.637	0.736	0.041
	2010	2479	63242	25.51	0.607	0.722	0.033
Buenos Aires	1980	215	3392	15.78	0.804	0.802	0.293
	1990	515	7071	13.73	0.634	0.734	0.148
	2000	1100	28515	25.92	0.654	0.741	0.069
	2010	1618	27120	16.76	0.669	0.746	0.069
Calcutta	1980	146	794	5.44	0.697	0.768	0.219
	1990	207	1655	8.00	0.626	0.735	0.208
	2000	166	2365	14.25	0.650	0.738	0.157
	2010	419	4246	10.13	0.571	0.710	0.095
Caltech	1980	1224	75433	61.63	0.671	0.751	0.047
	1990	1522	85480	56.16	0.654	0.743	0.046
	2000	2228	153289	68.80	0.650	0.742	0.034
	2010	2951	115107	39.01	0.642	0.737	0.028
Cambridge	1980	1353	65699	48.56	0.697	0.762	0.064
	1990	2273	118113	51.96	0.715	0.770	0.077
	2000	4062	224825	55.35	0.674	0.752	0.048
	2010	5303	182323	34.38	0.651	0.741	0.034
Chicago	1980	1307	63672	48.72	0.646	0.742	0.047
	1990	1586	91471	57.67	0.656	0.746	0.055
	2000	2045	134786	65.91	0.664	0.747	0.039
	2010	3285	114632	34.90	0.666	0.747	0.031
Cologne	1980	586	11663	19.90	0.680	0.755	0.123
	1990	751	20820	27.72	0.712	0.768	0.095
	2000	1298	46015	35.45	0.644	0.740	0.060
	2010	1727	37389	21.65	0.607	0.723	0.050
Columbia	1980	1432	73600	51.40	0.661	0.747	0.051
	1990	2021	123108	60.91	0.655	0.743	0.050
	2000	2796	188207	67.31	0.644	0.741	0.032
	2010	4906	155992	31.80	0.632	0.733	0.031
Delhi	1980	397	2710	6.83	0.645	0.741	0.141
	1990	238	2486	10.45	0.675	0.757	0.185
	2000	278	4499	16.18	0.667	0.749	0.104
	2010	835	11344	13.59	0.615	0.724	0.096
Edinburgh	1980	723	26317	36.40	0.721	0.772	0.069
	1990	1095	42946	39.22	0.654	0.742	0.070
	2000	1783	91050	51.07	0.652	0.742	0.044
	2010	2959	93173	31.49	0.648	0.740	0.031
Gottingen	1980	728	13058	17.94	0.644	0.739	0.109
	1990	966	38997	40.37	0.807	0.811	0.087
	2000	1442	55161	38.25	0.657	0.744	0.057
	2010	1993	48576	24.37	0.633	0.734	0.043
Groningen	1980	537	17216	32.06	0.620	0.730	0.054
	1990	937	36115	38.54	0.642	0.737	0.037
	2000	1472	70664	48.01	0.612	0.725	0.026
	2010	2992	82907	27.71	0.590	0.717	0.019
Harvard	1980	4517	240905	53.33	0.695	0.761	0.144
	1990	6150	440076	71.56	0.712	0.769	0.112
	2000	8732	710598	81.38	0.667	0.750	0.067
	2010	13446	474280	35.27	0.641	0.738	0.057
Heidelberg	1980	6240	239840	38.44	0.638	0.738	0.029
	1990	5630	215294	38.24	0.640	0.739	0.029
	2000	4709	181175	38.47	0.645	0.741	0.031
	2010	3200	122355	38.24	0.647	0.742	0.035
Helsinki	1980	6990	266778	38.17	0.634	0.736	0.029
	1990	9090	352316	38.76	0.627	0.733	0.028
	2000	10540	411872	39.08	0.626	0.733	0.027
	2010	11106	429641	38.69	0.626	0.733	0.027
HUJ	1980	1126	27316	24.26	0.633	0.732	0.052
	1990	1220	39586	32.45	0.639	0.739	0.050
	2000	1760	77577	44.08	0.656	0.743	0.039
	2010	1742	36882	21.17	0.594	0.717	0.041
IISC	1980	406	4908	12.09	0.715	0.771	0.195
	1990	543	9045	16.66	0.699	0.764	0.151
	2000	826	22600	27.36	0.657	0.746	0.074
	2010	1537	19963	12.99	0.606	0.722	0.076

Institutions	Year	Papers	Citations	$\langle c \rangle$	g	k	f_0
Kyoto	1980	1861	47153	25.34	0.651	0.743	0.074
	1990	2816	84563	30.03	0.662	0.747	0.063
	2000	4541	175219	38.59	0.668	0.749	0.051
	2010	5283	90834	17.19	0.619	0.728	0.065
Landau	1980	94	8136	86.55	0.862	0.859	0.064
	1990	124	3856	31.10	0.745	0.797	0.169
	2000	232	5961	25.69	0.790	0.806	0.168
	2010	256	2546	9.95	0.619	0.733	0.152
Leiden	1980	2100	33516	15.96	0.612	0.726	0.075
	1990	2550	43562	17.08	0.617	0.727	0.067
	2000	2950	50028	16.96	0.616	0.727	0.069
	2010	3650	63353	17.36	0.617	0.727	0.064
Leuven	1980	2816	84563	30.03	0.662	0.747	0.063
	1990	900	29257	32.51	0.692	0.761	0.062
	2000	3600	148209	41.17	0.654	0.744	0.039
	2010	950	38051	40.05	0.638	0.737	0.033
Madras	1980	180	1363	7.57	0.666	0.754	0.183
	1990	150	1165	7.77	0.666	0.756	0.180
	2000	181	2608	14.41	0.622	0.728	0.144
	2010	318	3969	12.48	0.753	0.783	0.176
Manchester	1980	150	1165	7.77	0.666	0.756	0.180
	1990	318	3969	12.48	0.753	0.783	0.176
	2000	800	20192	25.24	0.665	0.747	0.044
	2010	4289	107033	24.96	0.622	0.728	0.033
Melbourne	1980	1150	35610	30.97	0.572	0.710	0.012
	1990	850	22241	26.17	0.595	0.717	0.028
	2000	1067	31538	29.56	0.606	0.719	0.023
	2010	4289	107033	24.96	0.622	0.728	0.033
MIT	1980	2040	114892	56.32	0.713	0.769	0.109
	1990	2957	184009	62.23	0.724	0.777	0.099
	2000	3524	258609	73.39	0.716	0.772	0.062
	2010	880	12826	14.57	0.687	0.759	0.138
Osaka	1980	1603	33261	20.75	0.624	0.732	0.069
	1990	2853	73988	25.93	0.703	0.764	0.116
	2000	450	14717	32.70	0.646	0.742	0.051
	2010	6199	90104	14.54	0.680	0.753	0.125
Oslo	1980	741	17956	24.23	0.658	0.744	0.113
	1990	883	23639	26.77	0.647	0.740	0.121
	2000	1410	46806	33.20	0.603	0.721	0.065
	2010	2883	54926	19.05	0.587	0.715	0.059
Oxford	1980	971	39763	40.95	0.647	0.742	0.048
	1990	1781	87390	49.07	0.692	0.761	0.076
	2000	3351	222433	66.38	0.687	0.756	0.046
	2010	5539	199266	35.98	0.665	0.747	0.034
Princeton	1980	1122	46206	41.18	0.757	0.788	0.217
	1990	1474	73535	49.89	0.743	0.784	0.196
	2000	1996	114847	57.54	0.714	0.767	0.139
	2010	2684	75697	28.20	0.683	0.753	0.136
SINP	1980	31	180	5.81	0.670	0.746	0.194
	1990	85	702	8.26	0.632	0.731	0.118
	2000	140	1576	11.26	0.648	0.741	0.157
	2010	229	2920	12.75	0.679	0.752	0.131
Stanford	1980	2463	144640	58.73	0.737	0.780	0.117
	1990	3559	199720	56.12	0.698	0.764	0.115
	2000	5541	393605	71.04	0.734	0.779	0.082
	2010	7522	226442	30.10	0.679	0.755	0.082
Stockholm	1980	410	11393	27.79	0.695	0.762	0.124
	1990	648	22535	34.78	0.664	0.752	0.094
	2000	521	25832	49.58	0.685	0.756	0.060
	2010	312	8346	26.75	0.698	0.762	0.090
TAU	1980	1145	26937	23.53	0.718	0.770	0.114
	1990	1761	40134	22.79	0.679	0.751	0.114
	2000	2657	77106	29.02	0.663	0.746	0.074
	2010	3402	55252	16.24	0.657	0.744	0.103
TIFR	1980	163	2042	12.53	0.699	0.765	0.153
	1990	322	5256	16.32	0.745	0.780	0.196
	2000	437	12043	27.56	0.736	0.774	0.114
	2010	578	13803	23.88	0.747	0.778	0.104
Tokyo	1980	2595	60693	23.39	0.666	0.748	0.124
	1990	4383	121428	27.70	0.677	0.754	0.094
	2000	7734	267430	34.58	0.676	0.752	0.079
	2010	8980	164182	18.28	0.655	0.743	0.085
Toronto	1980	2568	80164	31.22	0.771	0.793	0.220
	1990	3613	116662	32.29	0.714	0.769	0.184
	2000	5185	246853	47.61	0.684	0.756	0.123
	2010	8880	207596	23.38	0.649	0.739	0.091
Yale	1980	2400	99265	41.36	0.716	0.770	0.202
	1990	2940	165230	56.20	0.725	0.774	0.162
	2000	4707	249190	52.94	0.723	0.773	0.163
	2010	6588	156268	23.72	0.684	0.756	0.143
Zurich	1980	712	16173	22.71	0.718	0.773	0.154
	1990	844	28233	33.45	0.684	0.756	0.120
	2000	1991	97451	48.95	0.661	0.748	0.065
	2010	3423	87603	25.59	0.629	0.729	0.059

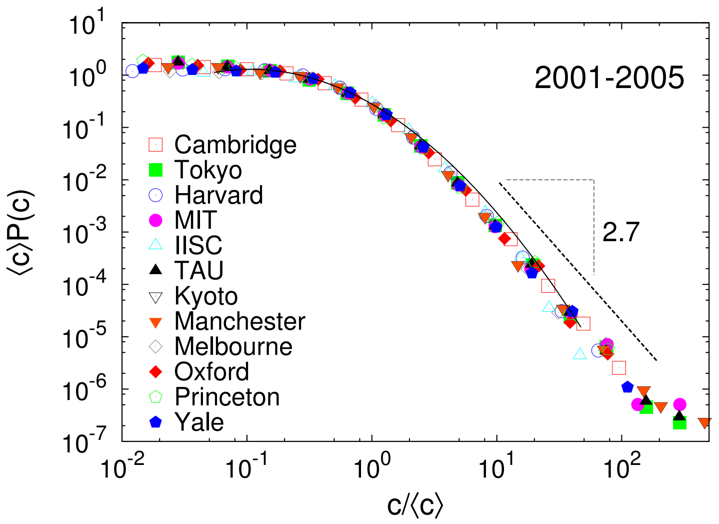

S1 Fig. B: **Probability distribution of citations for academic institutions, 2001-2005**
Probability distribution $P(c)$ of citations c rescaled by average number of citations $\langle c \rangle$ to publications from the period 2001-2005 for several academic institutions. Most of the range of the data fit well to a lognormal function with $\mu = -0.60 \pm 0.04$, $\sigma = 1.28 \pm 0.02$. but the highest citations fit to power law, with $\alpha = 2.7 \pm 0.3$.

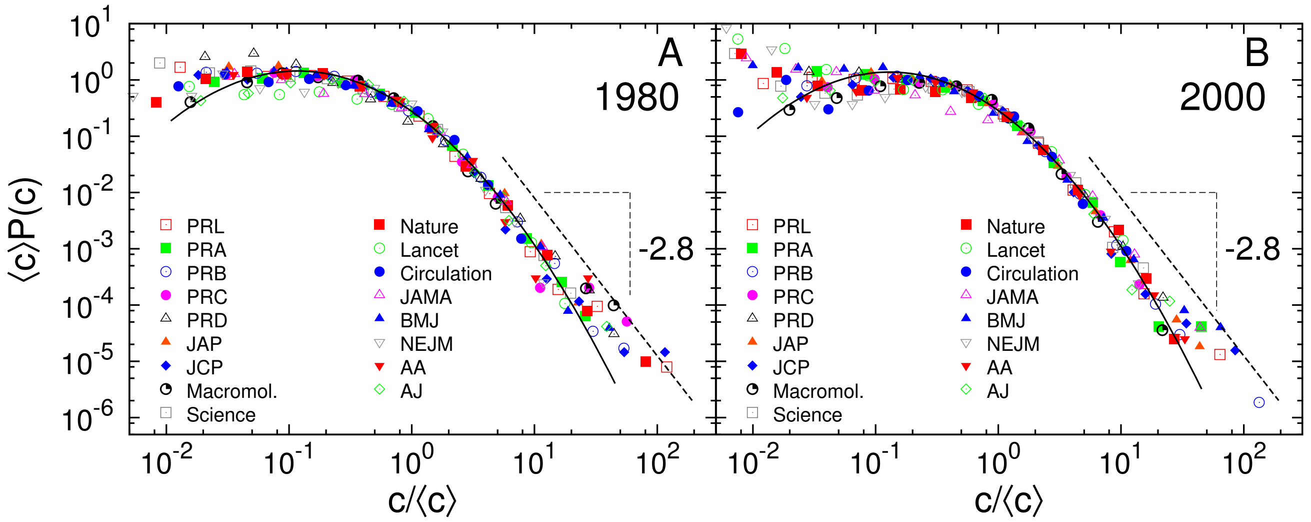

S1 Fig. C: **Probability distribution of citations for journals for 1980, 2000.**
Probability distribution $P(c)$ of citations c rescaled by average number of citations $\langle c \rangle$ to publications from 2 different years, (A) 1980 and (B) 2000 for several journals. The most of the range fits to a lognormal with $\mu = -0.75 \pm 0.02$, $\sigma = 1.18 \pm 0.02$ for 1980 and $\mu = -0.72 \pm 0.02$, $\sigma = 1.15 \pm 0.03$ for 2000. The largest citations for both the classes seem to follow a power law: $c^{-\alpha}$, with $\alpha = 2.8 \pm 0.4$ for 1980 and $\alpha = 2.8 \pm 0.3$ for 2000.

S1 TABLE D: **Academic journals: papers, citations etc.**

Table showing data for number of papers, total number of citations, average citation per paper $\langle c \rangle$, Gini (g) index, k index, and the fraction of uncited papers f_0 from different journals for several years.

Journals	Year	papers	citations	$\langle c \rangle$	g	k	f_0
Astronomy Astrophys.	1980	728	20594	28.29	0.636	0.734	0.026
	1990	909	27208	29.93	0.577	0.715	0.019
	2000	1412	51354	36.37	0.558	0.704	0.018
	2010	1916	40226	20.99	0.564	0.704	0.027
Astrophys. J.	1980	1223	64099	52.41	0.550	0.701	0.008
	1990	1517	72110	47.53	0.533	0.696	0.010
	2000	2388	136940	57.35	0.547	0.701	0.003
	2010	2501	73646	29.45	0.506	0.685	0.007
Biochem.	1980	935	55002	58.83	0.486	0.674	0.002
	1990	1510	104554	69.24	0.509	0.684	0.001
	2000	1823	79977	43.87	0.435	0.656	0.001
	2010	1142	18512	16.21	0.436	0.654	0.007
BMJ	1980	809	26008	32.15	0.676	0.757	0.080
	1990	626	31949	51.04	0.692	0.763	0.054
	2000	613	61513	100.35	0.709	0.769	0.038
	2010	293	14339	48.94	0.507	0.682	0.010
Circulation	1980	412	32786	79.58	0.555	0.704	0.022
	1990	541	62367	115.28	0.571	0.713	0.004
	2000	989	130463	131.91	0.528	0.693	0.004
	2010	554	41659	75.20	0.492	0.675	0.004
CPL	1980	1067	31538	29.56	0.606	0.719	0.023
	1990	1166	34366	29.47	0.627	0.730	0.025
	2000	1487	47478	31.93	0.579	0.713	0.011
	2010	1013	10966	10.83	0.525	0.687	0.051
Eur. J. Biochem.	1980	753	35476	47.11	0.545	0.697	0.005
	1990	789	35456	44.94	0.531	0.693	0.009
	2000	836	34696	41.50	0.514	0.683	0.004
	2010	447	9702	21.70	0.545	0.698	0.022
Inor. Chem.	1980	823	31391	38.14	0.459	0.666	0.001
	1990	1082	39076	36.11	0.476	0.672	0.004
	2000	941	36648	38.95	0.466	0.668	0.002
	2010	1416	31953	22.57	0.447	0.662	0.007
JAMA	1980	503	13999	27.83	0.675	0.753	0.205
	1990	743	48102	64.74	0.762	0.787	0.353
	2000	699	79037	113.07	0.757	0.788	0.330
	2010	535	34563	64.60	0.723	0.772	0.187
JAP	1980	1108	34304	30.96	0.668	0.754	0.046
	1990	2571	57132	22.22	0.638	0.739	0.054
	2000	2941	80656	27.42	0.613	0.727	0.027
	2010	3892	40286	10.35	0.511	0.685	0.053
J. Chem. Phys.	1980	1849	101604	54.95	0.651	0.739	0.016
	1990	1958	95959	49.01	0.582	0.711	0.012
	2000	2526	102525	40.59	0.579	0.710	0.005
	2010	2137	32872	15.38	0.522	0.686	0.022
JMMM	1980	907	7467	8.23	0.631	0.735	0.128
	1990	852	8734	10.25	0.653	0.744	0.131
	2000	802	12714	15.85	0.584	0.714	0.062
	2010	740	6382	8.62	0.570	0.708	0.099
J. Org. Chem.	1980	1232	38706	31.42	0.513	0.687	0.004
	1990	1162	44254	38.08	0.494	0.680	0.001
	2000	1396	53495	38.32	0.442	0.659	0.002
	2010	1201	25166	20.95	0.417	0.649	0.005
JPA	1980	340	7800	22.94	0.752	0.790	0.106
	1990	470	7507	15.97	0.625	0.735	0.085
	2000	644	8758	13.60	0.592	0.722	0.079
	2010	959	6545	6.82	0.573	0.707	0.115
Lancet	1980	595	38780	65.18	0.650	0.736	0.229
	1990	476	67331	141.45	0.604	0.721	0.048
	2000	822	109177	132.82	0.642	0.739	0.050
	2010	271	54158	199.85	0.463	0.670	0.000

Journals	Year	papers	citations	$\langle c \rangle$	g	k	f_0
Langmuir	1990	285	12679	44.49	0.589	0.716	0.014
	2000	1476	72398	49.05	0.528	0.694	0.003
	2010	2664	55540	20.85	0.460	0.665	0.007
Macromol.	1980	315	20018	63.55	0.642	0.737	0.029
	1990	863	42799	49.59	0.567	0.710	0.008
	2000	1373	69158	50.37	0.499	0.682	0.001
	2010	1365	38215	28.00	0.472	0.668	0.006
Nature	1980	1502	181108	120.58	0.637	0.736	0.007
	1990	1391	315723	226.98	0.676	0.751	0.180
	2000	1517	464531	306.22	0.668	0.746	0.146
	2010	1012	163098	161.16	0.547	0.698	0.111
NEJM	1980	360	67263	186.84	0.518	0.686	0.003
	1990	374	111199	297.32	0.506	0.683	0.000
	2000	379	142799	376.78	0.603	0.720	0.055
	2010	342	77333	226.12	0.576	0.706	0.029
Physica A	1980	195	3298	16.91	0.551	0.700	0.067
	1990	402	6691	16.64	0.653	0.748	0.112
	2000	620	10776	17.38	0.649	0.744	0.077
	2010	617	5217	8.46	0.587	0.718	0.100
Physica B	1990	1187	6787	5.72	0.632	0.732	0.190
	2000	2630	13378	5.09	0.647	0.740	0.220
	2010	1058	7139	6.75	0.558	0.702	0.127
Physica C	1990	608	16779	27.60	0.586	0.715	0.016
	2000	1621	8481	5.23	0.664	0.748	0.240
	2010	897	2528	2.82	0.658	0.744	0.309
PRA	1980	624	25452	40.79	0.609	0.724	0.024
	1990	1859	57309	30.83	0.603	0.724	0.033
	2000	1410	42545	30.17	0.624	0.729	0.044
	2010	2858	39628	13.87	0.519	0.687	0.031
PRB	1980	1354	64235	47.44	0.648	0.743	0.018
	1990	3390	161651	47.68	0.649	0.741	0.017
	2000	4756	172663	36.30	0.602	0.722	0.025
	2010	6049	108383	17.92	0.528	0.692	0.021
PRC	1980	619	19945	32.22	0.653	0.744	0.027
	1990	703	16018	22.79	0.617	0.728	0.033
	2000	832	20521	24.66	0.569	0.709	0.040
	2010	1013	18071	17.84	0.545	0.697	0.032
PRD	1980	775	37491	48.38	0.763	0.797	0.081
	1990	1007	34899	34.66	0.681	0.759	0.049
	2000	2020	70517	34.91	0.613	0.728	0.040
	2010	2931	56035	19.12	0.532	0.693	0.028
PRE	2000	2034	58313	28.67	0.584	0.715	0.022
	2010	2310	26163	11.33	0.492	0.678	0.039
PRL	1980	1194	92238	77.25	0.670	0.746	0.047
	1990	1643	163084	99.26	0.604	0.724	0.003
	2000	3046	250871	82.36	0.589	0.717	0.006
	2010	3105	115955	37.34	0.493	0.679	0.004
Science	1980	1021	115716	113.34	0.635	0.738	0.032
	1990	1061	237803	224.13	0.663	0.745	0.168
	2000	1053	343455	326.17	0.614	0.725	0.092
	2010	974	135833	139.46	0.529	0.692	0.089
Tetrahedron	1980	391	16696	42.70	0.709	0.771	0.013
	1990	684	19410	28.38	0.556	0.701	0.034
	2000	1092	33027	30.24	0.503	0.680	0.007
	2010	1179	16155	13.70	0.465	0.665	0.015

S1 TABLE E: **Elite journals, considering all citable documents: papers, citations etc.**
Table showing data for number of citable documents, total number of citations, average citation per paper $\langle c \rangle$, Gini (g) index, k index, as well as the fraction of uncited papers f_0 from different journals of the *Elite* class for several years.

Journals	Year	papers	citations	$\langle c \rangle$	g	k	f_0
BMJ	1980	3056	27798	9.10	0.886	0.869	0.542
	1990	2824	37353	13.23	0.884	0.867	0.482
	2000	3263	71373	21.87	0.901	0.877	0.451
	2010	3412	19308	5.66	0.900	0.883	0.601
Circulation	1980	1895	35986	18.99	0.861	0.861	0.387
	1990	3830	70278	18.35	0.907	0.891	0.478
	2000	5574	137302	24.63	0.906	0.887	0.628
	2010	6675	46645	6.99	0.951	0.931	0.827
JAMA	1980	1440	20784	14.43	0.794	0.812	0.386
	1990	1738	54443	31.33	0.876	0.862	0.472
	2000	1696	86713	51.13	0.869	0.862	0.381
	2010	1425	36235	25.43	0.863	0.857	0.435
Lancet	1980	2981	56515	18.96	0.822	0.829	0.378
	1990	3230	94008	29.10	0.846	0.842	0.344
	2000	3367	123585	36.70	0.870	0.863	0.365
	2010	1743	62028	35.59	0.876	0.873	0.368
Nature	1980	2892	185484	64.14	0.799	0.809	0.336
	1990	3606	318090	88.22	0.857	0.847	0.435
	2000	3612	330512	91.50	0.856	0.847	0.434
	2010	2577	177161	68.75	0.791	0.809	0.302
NEJM	1980	1791	77780	43.43	0.858	0.855	0.376
	1990	1684	122750	72.89	0.854	0.851	0.348
	2000	1561	155490	99.61	0.874	0.864	0.336
	2010	1753	93609	53.40	0.867	0.861	0.345
Science	1980	1669	117642	70.49	0.765	0.795	0.217
	1990	2178	243190	111.66	0.826	0.829	0.354
	2000	2575	363418	141.13	0.816	0.823	0.260
	2010	2439	154194	63.22	0.762	0.795	0.243

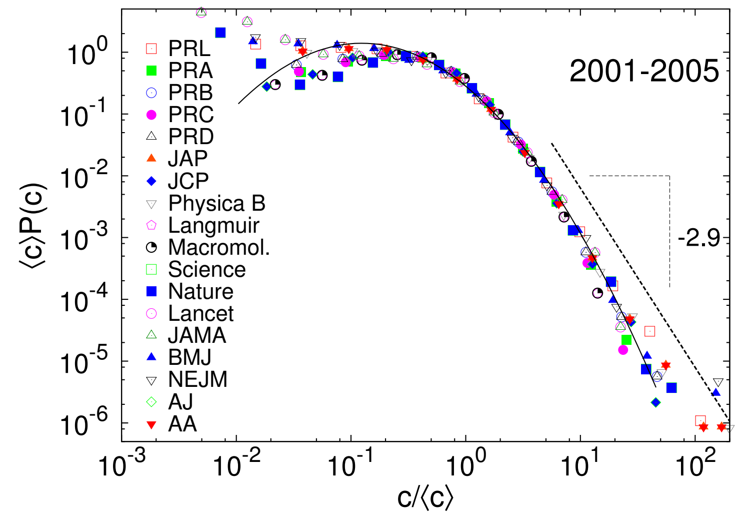

S1 Fig. D: **Probability distribution of citations for journals for 2001-2005.**
Probability distribution $P(c)$ of citations c rescaled by average number of citations $\langle c \rangle$ to publications from 2001-2005 for several journals. The most of the range fits to a lognormal with $\mu = -0.73 \pm 0.02$, $\sigma = 1.16 \pm 0.02$, while the largest citations seem to follow a power law: $c^{-\alpha}$, with $\alpha = 2.9 \pm 0.2$.

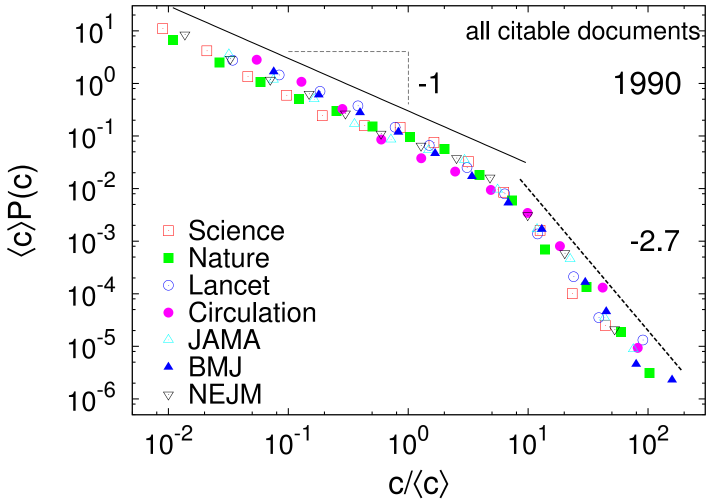

S1 Fig. E: **Rescaled probability distributions of citation to all citable documents for several journals of the Elite class for 1990**
Probability distribution $P(c)$ of citations c rescaled by average number of citations $\langle c \rangle$ to publications from 1990 for all citable documents of several academic journals in the *Elite* class: The scaling distribution is such that $\langle c \rangle P(c) \sim (c/\langle c \rangle)^{-b}$ with $b \simeq 1$, for the lower range of c. The largest citations fit well to a power law: $c^{-\alpha}$, with $\alpha = 2.7 \pm 0.4$.

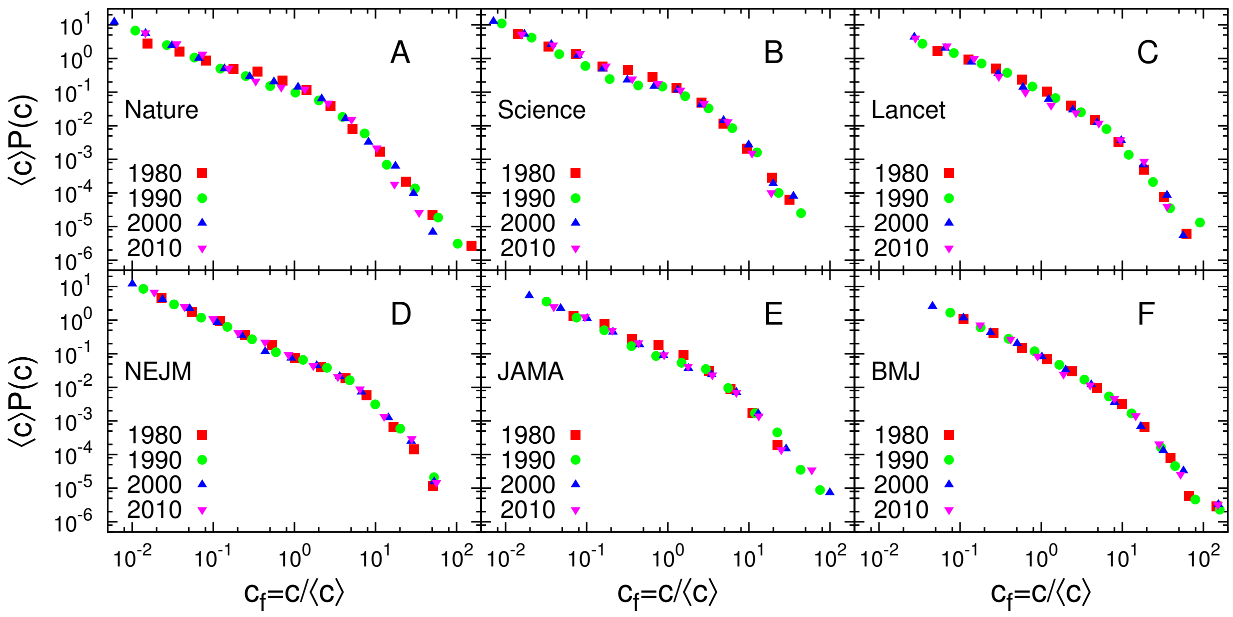

S1 Fig. F: **Rescaled probability distributions of citation to all citable documents for several journals of the Elite class for different years.**
Probability distribution $P(c)$ of citations c rescaled by average number of citations $\langle c \rangle$ to publications from 1980, 1990, 2000 and 2010 for all citable documents of several academic journals in the *Elite* class.

S1 TABLE F: **Power law exponents.**
Average power law exponents for the highest cited publications for different years (1980, 1990, 2000 and 2010), for all institutions and journals considered.

Year	Institutions	Journals
1980	2.8 ± 0.2	2.8 ± 0.4
1990	2.8 ± 0.2	2.9 ± 0.3
2000	2.8 ± 0.1	2.8 ± 0.3
2010	2.8 ± 0.2	3.0 ± 0.4

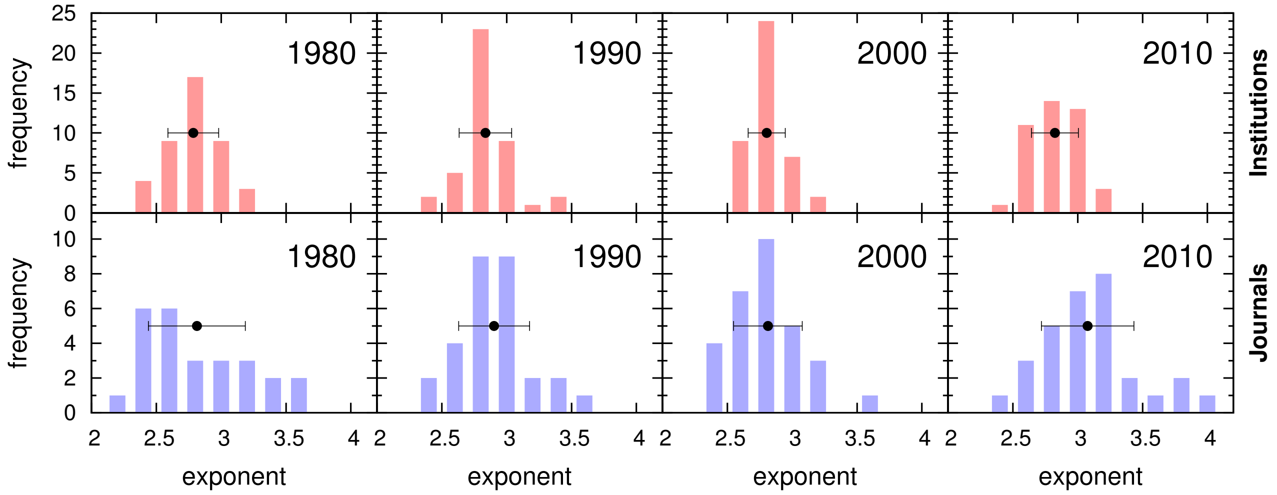

S1 Fig. G: **Histograms for power law exponents.**
Histograms of power law exponents of the highest cited papers measured from individual data sets, their average along with error bar. Data is shown for 4 different years: 1980, 1990, 2000 and 2010, for institutions and journals.
